# TAp73 and ΔTAp73 isoforms show cell-type specific distributions and alterations in cancer

**DOI:** 10.1038/s41598-024-80927-9

**Published:** 2024-12-02

**Authors:** Vaclav Hrabal, Michaela Stenckova, Filip Zavadil Kokas, Petr Muller, Rudolf Nenutil, Borivoj Vojtesek, Philip J. Coates

**Affiliations:** 1 Research Center for Applied Molecular Oncology (RECAMO), Masaryk Memorial Cancer Institute, Zluty kopec 7, Brno, 656 53 Czech Republic; 2 Department of Experimental Biology, Faculty of Science, Masaryk University, Brno, Czech Republic; 3 Department of Pathology, Masaryk Memorial Cancer Institute, Brno, Czech Republic

**Keywords:** p73 isoforms, Multiciliated cells, Fallopian tube, Endometrium, Squamous epithelial stem cells, Cervical cancer, Cancer, Cell biology, Medical research

## Abstract

*TP73* is a member of the *TP53* gene family and produces N- and C-terminal protein isoforms through alternative promoters, alternative translation initiation and alternative splicing. Most notably, p73 protein isoforms may either contain a p53-like transactivation domain (TAp73 isoforms) or lack this domain (ΔTAp73 isoforms) and these variants have opposing or independent functions. To date, there is a lack of well-characterised isoform-specific p73 antibodies. Here, we produced polyclonal and monoclonal antibodies to N-terminal p73 variants and the C-terminal p73α isoform, the most common variant in human tissues. These reagents show that TAp73 is a marker of multiciliated epithelial cells, while ΔTAp73 is a marker of non-proliferative basal/reserve cells in squamous epithelium. We were unable to detect ΔNp73 variant proteins, in keeping with recent data that this is a minor form in human tissues. Most cervical squamous cell carcinomas (79%) express p73α, and the distribution of staining in basal cells correlated with lower tumour grade. TAp73 was found in 17% of these tumours, with a random distribution and no association with clinicopathological features. These data indicate roles for ΔTAp73 in maintaining a non-proliferative state of undifferentiated squamous epithelial cells and for TAp73 in the production of differentiated multiciliated cells.

**Supplementary Information:**

The online version contains supplementary material available at 10.1038/s41598-024-80927-9.

## Introduction

p73, together with p53 and p63, belongs to the p53 family of transcription factors. p53 was the first family member identified, discovered in 1979^1,2^, and p63^3^ and p73^4^ were first described in 1997. p53 is a well-known tumour suppressor that functions in growth arrest, apoptosis and senescence after cellular stress^5^. Based on sequence homology, it was presumed that p63 and p73 have similar roles to p53^6,7^. However, the main function of p63 is regulation of epithelial development and morphogenesis^8^, whereas p73 is involved in nervous system development, regulation of multicilliogenesis, and angiogenesis^9^. In contrast to p53, p63 and p73 are rarely mutated in cancer, and the p63 isoform ΔNp63 is oncogenic and is often overexpressed in human squamous cell carcinomas^10–12^.

**Fig. 1:**
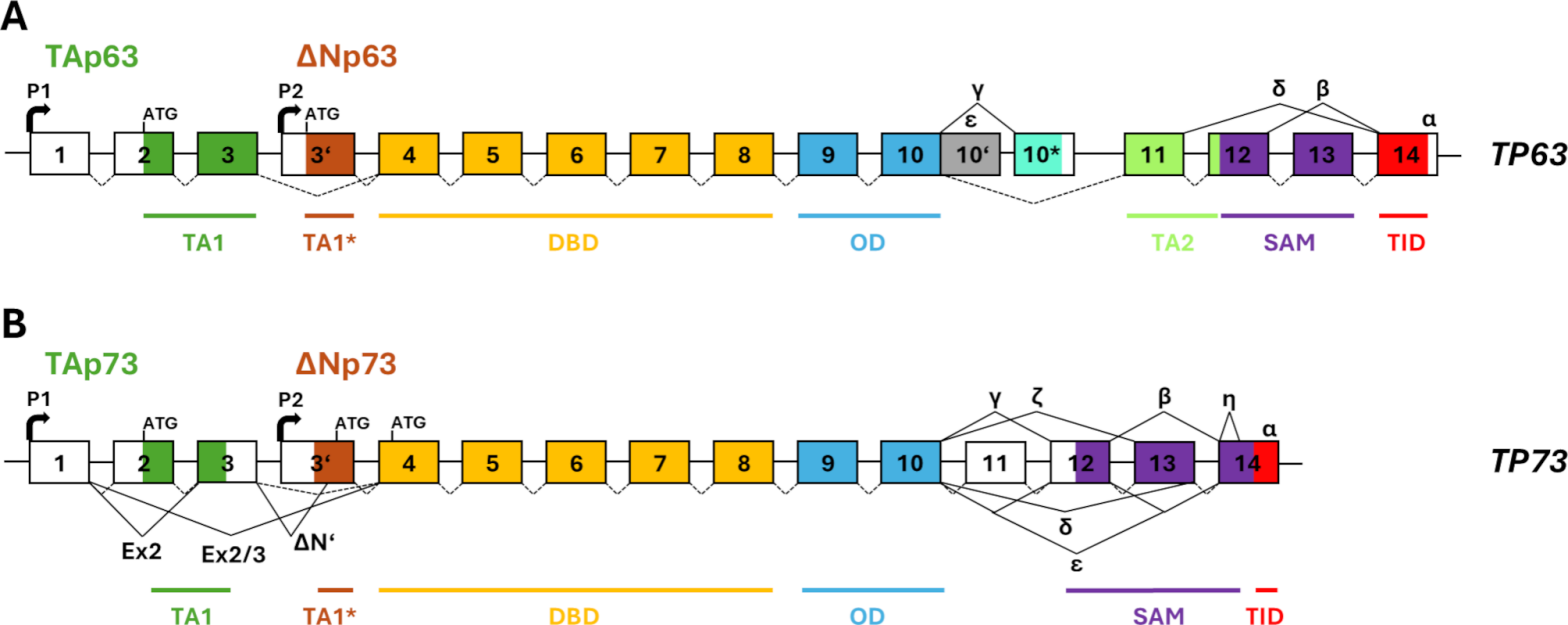
Schematic representation of *TP63* and *TP73* gene structure. (**A**) Transcription from promoter P1 and P2 of *TP63* in combination with multiple possible 3ʹ-end splice variants may give rise to at least 10 mRNAs: TAp63α, TAp63β, TAp63γ, TAp63δ, TAp63ε, ΔNp63α, ΔNp63β, ΔNp63γ, ΔNp63δ, ΔNp63ε. (**B**) *TP73* is more complex and 5ʹ-end variability is increased by additional splice variants Ex2, Ex2/3 and ΔNʹ. Alternative initiation of translation of transcripts at the first in-frame ATG of exon 4 may also result in another set of ΔTAp73 isoforms. There are also more 3ʹ-end splice variants: α,β,γ,δ,ε,ζ,η. TA1, TA1*, TA2, sequences important for transactivation. *DBD* DNA binding domain, *OD* oligomerisation domain, *SAM* sterile alpha motif, *ID* inhibitory domain.

p73 is encoded by the *TP73* gene located on the short arm of chromosome 1 (1p36)^4^. By analogy with *TP63*, *TP73* is proposed to contain two promoters and in combination with alternative splicing can produce several protein isoforms that differ in their N- and C-terminal amino acid sequences^13,14^. Transcription from the upstream promoter, P1, gives rise to the longer N-terminal TAp73 isoforms that contain a transactivation domain (TAD) with high homology to the p53 and p63 TADs. Alternatively spliced mRNA variants transcribed from P1 give rise to ΔEx2p73, ΔEx2/3p73 and ΔN’p73 transcripts that lack parts of the TAD coding sequences, collectively termed ΔTAp73 isoforms. In addition, ΔNp73 is transcribed from a downstream promoter, P2, and therefore lacks exons 1 to 3 that encode the TAD but contains an alternative exon (exon 3’) that encodes a unique short amino acid sequence, similar to the mechanism employed to produce the ΔNp63 variant of *TP63*^15^ (Fig. 1). Analysis of human tissue RNA-seq data from the GTEx Project^16,17^ predicted a further ΔTAp73 product encoded by exons 4 to 14, with little evidence for expression of the ΔNp73 exon3’ or Δexon2/3 variants^14,18^.

In addition to these N-terminal protein isoforms, alternative splicing of 3’-end exons may produce at least seven protein variants that differ at the C-terminus; α, β, γ, δ, ε, ζ, η^13^. However, RNA-Seq analyses indicate that p73α variants are the major isoforms present in human cells and tissues^14,18^.

Mice functionally deficient for all p73 isoforms exhibit profound defects, including hippocampal dysgenesis, hydrocephalus, chronic infections and inflammation, as well as abnormalities in pheromone sensory pathways^19^. Cross-species genomic analyses and functional rescue experiments identify TAp73 as a master transcriptional integrator of multiciliogenesis, and the lack of this process in *Trp73*-null mice can account for most if not all of their developmental defects and chronic inflammation^20–22^. TAp73 also influences cellular metabolism and energy production through transcriptional regulation of metabolic enzymes such as glutaminase-2 and glucose-6 phosphate dehydrogenase^23^. In contrast, ΔNp73 has a pro-survival role in discrete neuron types including Cajal–Retzius neurons and the choroid plexus^9^.

Using immunohistochemistry, p73α is present in the nuclei of basal and parabasal cells of squamous epithelium in the oesophagus, tonsils, skin, hair follicles and in the basal layer of cervix^24,25^. p73α was also observed in basal cells of columnar epithelium in the larynx and upper bronchi, in the transitional epithelium of the bladder, in glandular epithelial cells in breast and prostate, and in spermatogonia. Moderate staining of p73α was also observed in the parotid gland and in occasional cells in the colon^25^.

Those previous studies have not defined the specific p73 isoforms that are expressed by each cell type, and there is therefore a need for antibodies that recognise individual p73 isoforms or individual groups of isoforms. Another problem with detecting p53 family proteins is antibody cross-reactivity, where most p73 antibodies also bind to p63 or show non-specific binding to proteins outside the p53 family^26^, while the majority of monoclonal antibodies developed against p63 also cross-react with p73^27^. Here we have produced and characterised novel antibodies that specifically recognise TAp73, p73α, or ΔNp73 without cross-reactivity to other isoforms or to other p53 family proteins.

## Results

### Novel antibodies recognising ΔNp73, TAp73 and p73α isoforms

**Fig. 2:**
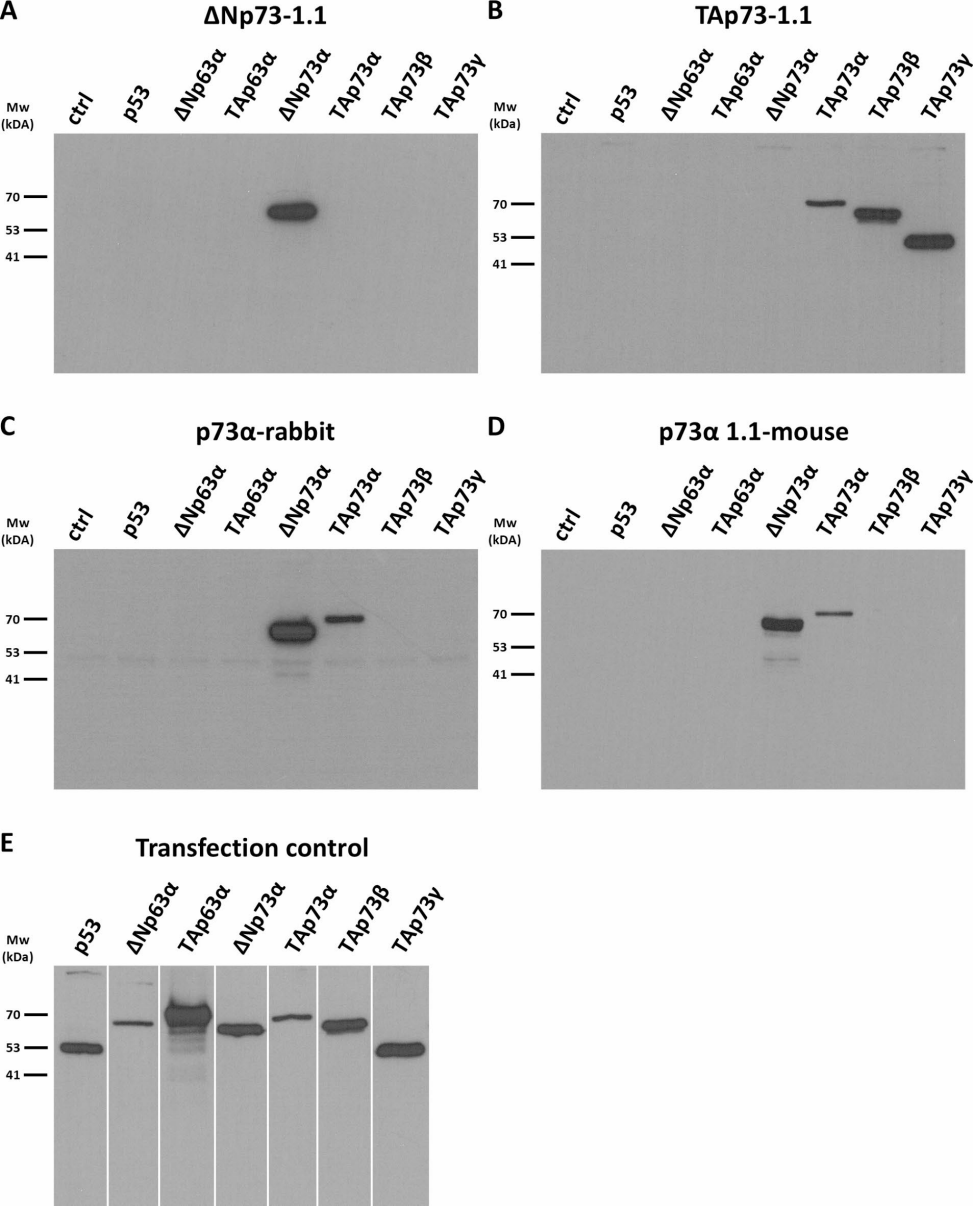
Western blot analysis of antibody specificity. H1299 cells were transiently transfected with plasmids coding for p53, p63 isoforms or p73 isoforms, and non-transfected H1299 cells were used as a negative control (ctrl). (**A**–**D**) Detection with antibodies recognising individual p73 isoforms: (**A**) ΔNp73-1.1 mouse monoclonal; (**B**) TAp73-1.1 recognising TAp73; (**C**) rabbit polyclonal p73α; (**D**) p73α-1.1 mouse monoclonal. Whole membranes are shown to reveal potential non-specific or cross-reactive bands. (**E**) To monitor successful transfection and protein expression, the membrane was cut into vertical strips representing each individual lane, and each lane was detected with antibodies that recognise the transfected protein. Original film images are shown in Supplementary Figure S1 online.

We developed polyclonal and monoclonal antibodies to different regions of p73. Altogether, seven monoclonal antibodies were chosen from primary screening. TAp73-1.1 recognises the p73 TAD recombinant protein; ΔNp73-1.1, ΔNp73-2.1 and ΔNp73-3.1 recognise the ΔNp73 specific peptide, and p73α-1.1, p73α-2.1 and p73α-3.1 each recognise the p73α specific peptide. These mouse monoclonal antibodies together with affinity purified rabbit polyclonal antibodies to ΔNp73 and p73α were tested against p73 isoforms and for cross-reactivity to p63 isoforms and p53 using Western blotting of lysates of H1299 cells transiently transfected with expression vectors for each protein (Fig. 2 and Supplementary Fig. S1 online). Interestingly, in these blots the levels of ΔNp73 are higher than TAp73, and TAp73β and TAp73γ are higher than TAp73α, which may relate to differential protein stability through the action of various ubiquitin E3 ligases that target different isoforms^14^. The mouse monoclonal antibodies ΔNp73-1.1, ΔNp73-2.1 and ΔNp73-3.1 recognise only ΔNp73 proteins, with no cross-reaction with TAp73, p53 or p63 isoforms (Fig. 2A). Similarly, affinity purified rabbit anti-ΔNp73 recognises ΔNp73 but does not bind to TAp73, TAp63, ΔNp63, or p53. TAp73-1.1 binds to TAp73α, β and γ isoforms and does not bind to either TAp63α or ΔNp63α, or to p53 (Fig. 2B). The affinity purified rabbit anti-p73α serum and the mouse monoclonal antibodies p73α-1.1, p73α-2.1 and p73α-3.1 all recognise TAp73α and ΔNp73α, but not p73β or p73γ isoforms, and show no cross-reaction with p63 or p53 (Fig. 2C and D).

### Evaluation for use in immunohistochemistry

**Fig. 3:**
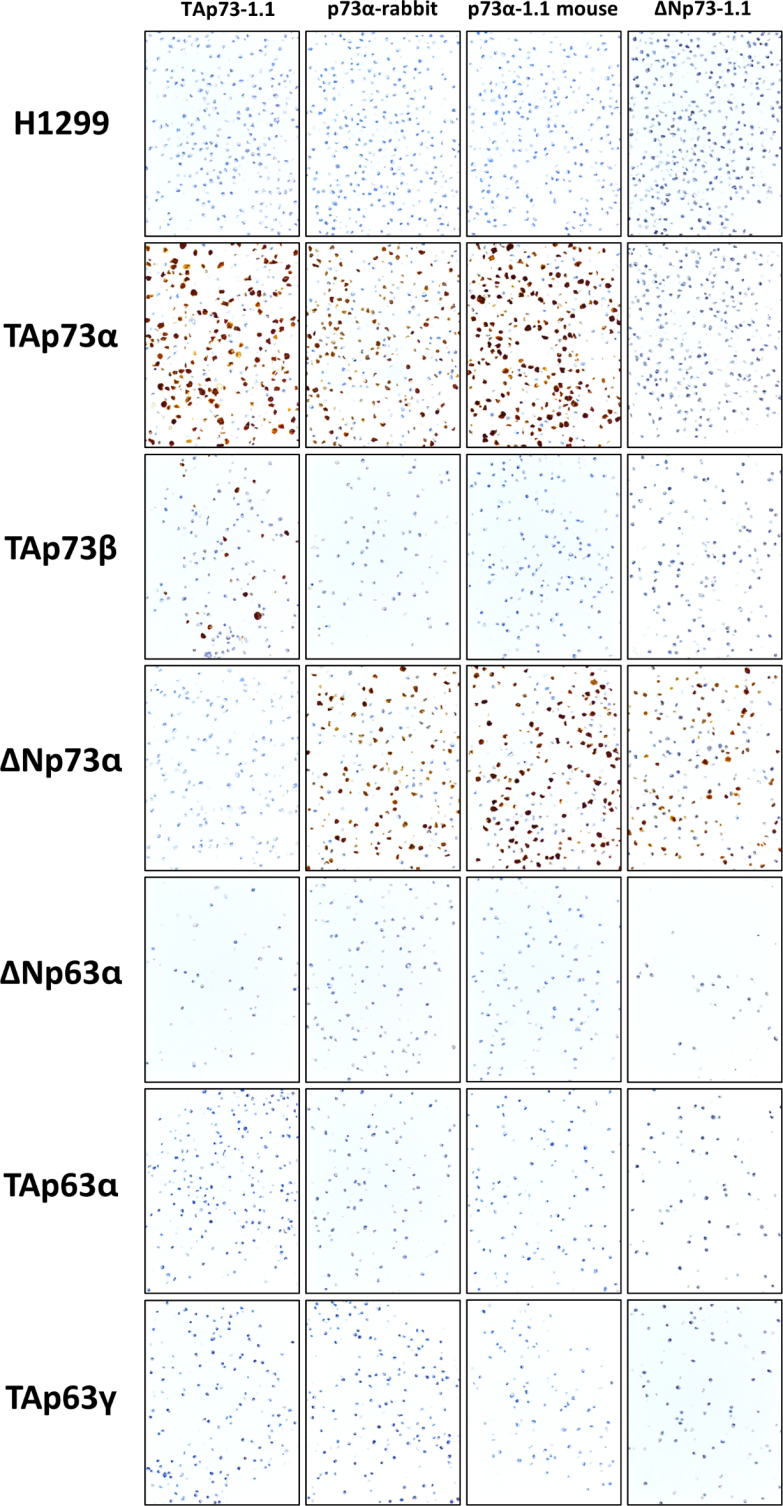
Immunocytochemical analysis of antibody specificity. Non-transfected H1299 cells or cells transfected with plasmids containing genes coding for p53 family isoforms (indicated down the left side of the figure) were formalin fixed and embedded in paraffin. Sections were immunocytochemically stained with the four antibodies indicated across the top of the figure (TAp73-1.1, affinity purified rabbit anti-p73α, mouse monoclonal p73α-1.1, and mouse monoclonal ΔNp73-1.1). Positive staining is seen as a brown precipitate and nuclei were counterstained with haemotoxylin (blue). Non-transfected H1299 cells were used as a negative control. Please note that transfections were performed transiently, therefore not all cells contain the expression plasmid.

To evaluate the applicability of these antibodies for immunostaining of formalin-fixed, paraffin-embedded samples and to confirm antibody specificity using this method, we immunostained sections of paraffin-embedded cell pellets of H1299 cells transiently transfected with plasmids carrying a range of p53 family proteins (TAp73α, TAp73β, ΔNp73α, ΔNp63α, TAp63α, TAp63γ). All antibodies specifically stained the nuclei of cells transfected with the appropriate protein isoform. TAp73-1.1 mouse monoclonal antibody stained the nuclei of cells transfected with TAp73 isoforms (TAp73α and TAp73β) but did not stain cells transfected with ΔNp73α. Affinity purified rabbit polyclonal p73α and the mouse monoclonal antibody p73α-1.1 stained only cells transfected with p73α isoforms (TAp73α and ΔNp73α) and not with TAp73β, while the monoclonal antibody ΔNp73-1.1 stained only cells transfected with ΔNp73α and did nor stain cells transfected with TAp73 isoforms. In addition, none of the p73 antibodies showed cross-reactivity with the corresponding p63 isoforms (Fig. 3), or with p53. These data are identical to the results of Western blotting shown in Fig. 2 and confirm the specificity of the reagents for detecting the corresponding p73 isoform in formalin-fixed, paraffin-embedded material.

### Phage display epitope mapping

**Fig. 4:**
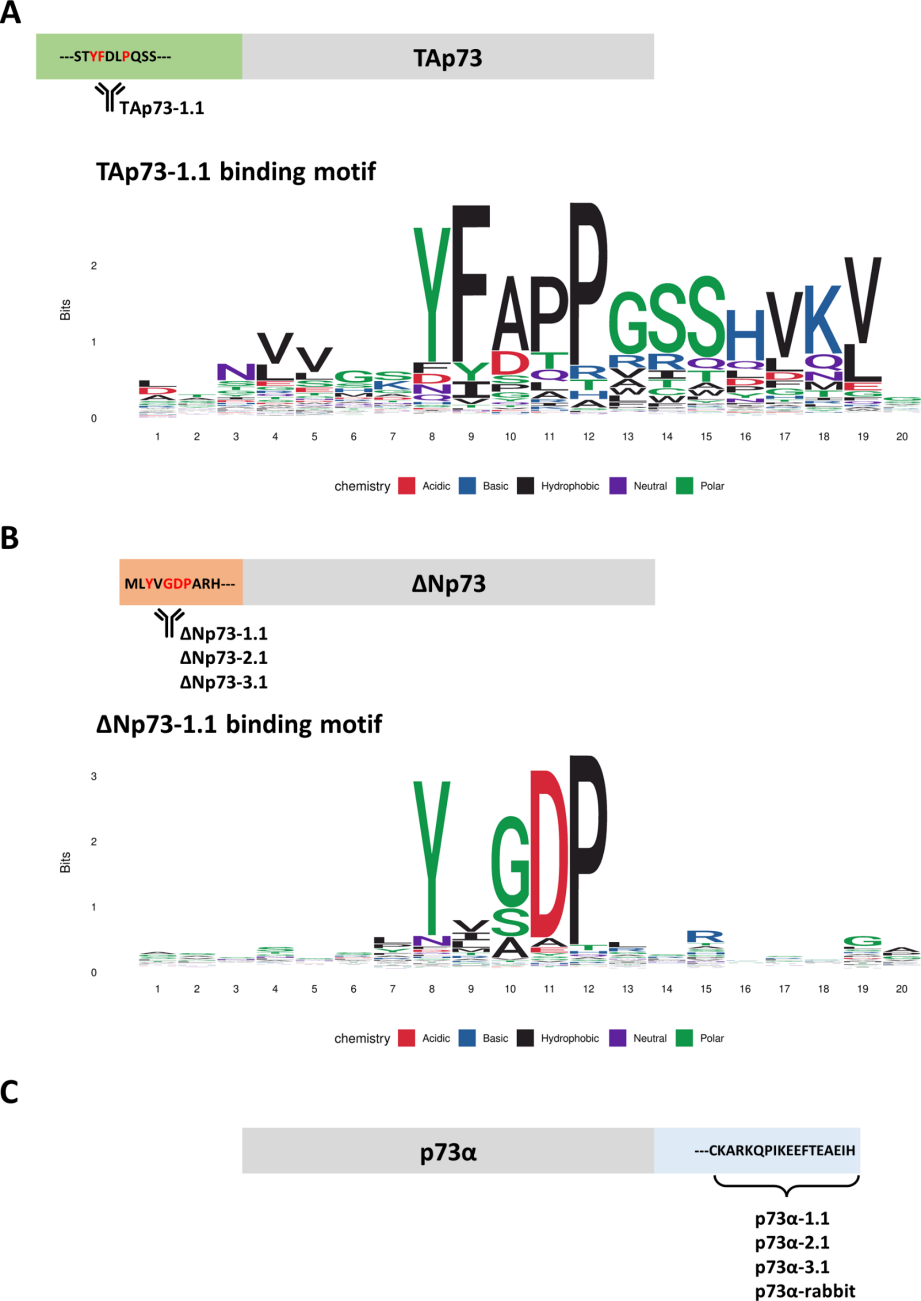
Representation of antibody binding sites and binding motifs acquired from phage display epitope mapping. (**A**) Schematic representation and WebLogo image showing the TAp73-1.1 binding motif result from phage display epitope mapping. (**B**) Schematic representation of the binding sites of ΔNp73-1.1, ΔNp73-2.1 and ΔNp73-3.1. The WebLogo image shows the ΔNp73-1.1 binding motif from the phage display. (**C**) Binding site of p73α-1.1, p73α-2.1, p73α-3.1 and p73α rabbit antibodies.

The TAp73-1.1 antibody was raised using the full sequence of the TA domain of TAp73 as immunogen. To identify the precise epitope within this sequence, we performed phage display epitope mapping, in which the antibody binds to and enriches individual phage that display 12-mer amino acid peptides that bind to the antibody. The resulting phage are then sequenced and analysed for enrichment of specific amino acid motifs. This process identified the sequence YFDLP, corresponding to amino acids 28–32 of TAp73 (Fig. 4A). We also mapped the epitope of ΔNp73-1.1 as YVGDP (amino acids 3–8 of ΔNp73) (Fig. 4B). The epitope of p73α antibodies lie within the extreme C-terminal region (Fig. 4C).

### p73 isoforms in normal tissues

**Fig. 5:**
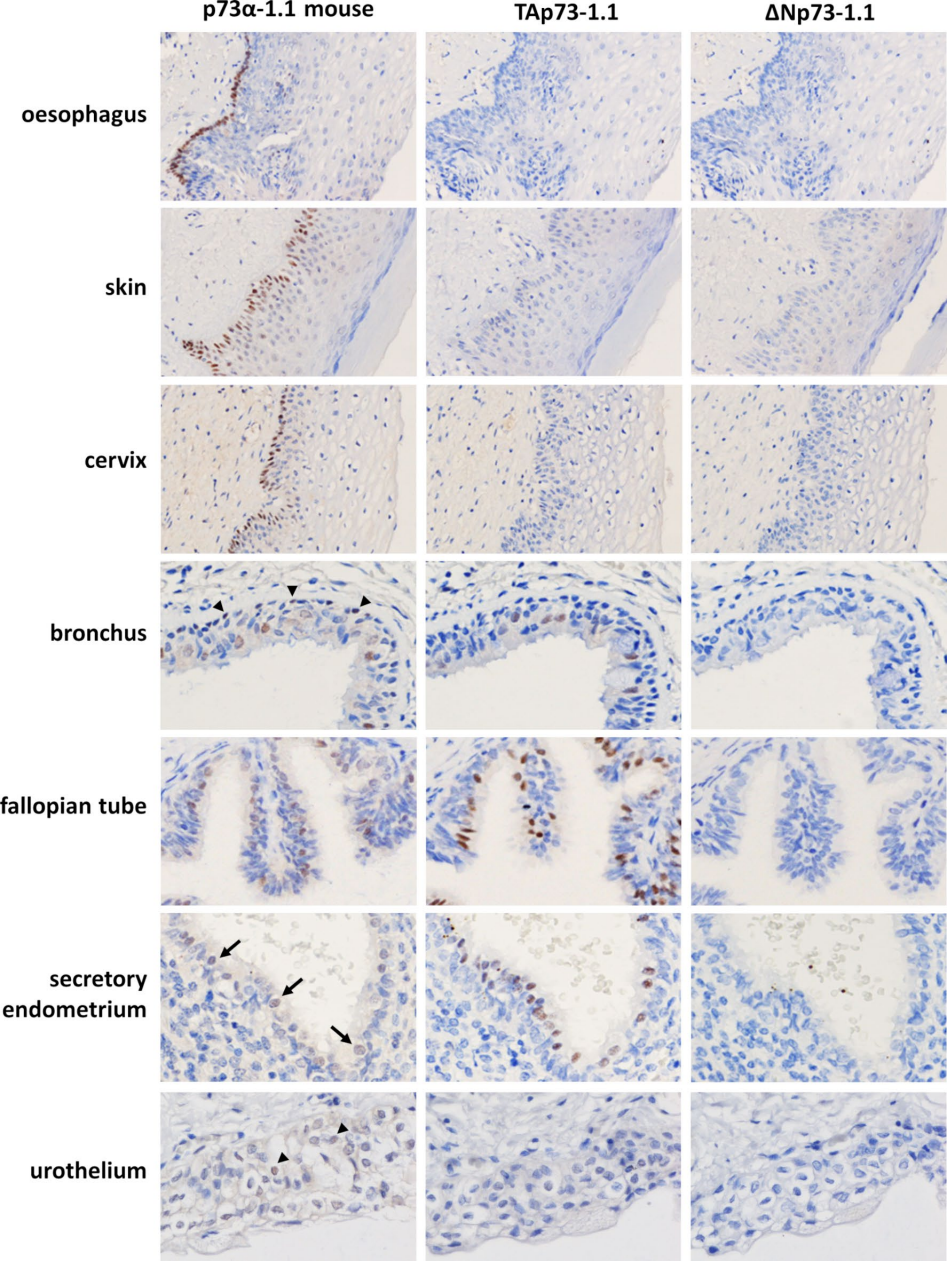
Immunohistochemical staining of normal tissues. FFPE tissue sections were immunostained with p73α-1.1, TAp73-1.1 and ΔNp73-1.1 antibodies (brown) and nuclei were counterstained with haemotoxylin (blue). Additional tissues are shown in Supplementary Figure S3 online.

Sections of formalin-fixed paraffin-embedded (FFPE) normal human tissues were stained with the newly developed mouse monoclonal and affinity purified rabbit polyclonal antibodies (Table 1). Staining with the mouse monoclonal antibody TAp73-1.1 was confined to the nuclei of multiciliated cells in the bronchus, fallopian tube and secretory endometrium, with no staining seen in the other tissues examined. These cells were also positive with monoclonal antibodies and affinity purified rabbit polyclonal to p73α. Staining for p73α was more widespread than TAp73, and was seen also in nuclei of the basal layer of squamous epithelial cells in the oesophagus, skin and cervix, and in basal cells in the bronchus. The same pattern of staining was seen using p73α-1.1, p73α-3.1 or affinity purified rabbit anti-p73α, while p73α-2.1 showed similar staining but exhibited high background (Supplementary Fig. S2 online). Further studies were therefore conducted using p73α-1.1 and affinity purified rabbit anti-p73α. Despite using high antibody concentrations (up to 3 µg/ml), none of the monoclonal or affinity purified polyclonal antibodies to ΔNp73 stained any cells in any of the tissues studied (Fig. 5), although they robustly recognise this isoform in FFPE cell blocks at the same antibody concentrations as the other p73 reagents developed here (0.1 µg/ml or lower). Figure 5 shows examples of tissues positive for either TAp73 or p73α. Tissues that are negative for all p73 isoforms are shown in Supplementary Figure S3 online.

### p73 co-localisation with p63 defines the basal cell layer in normal squamous epithelium

**Fig. 6:**
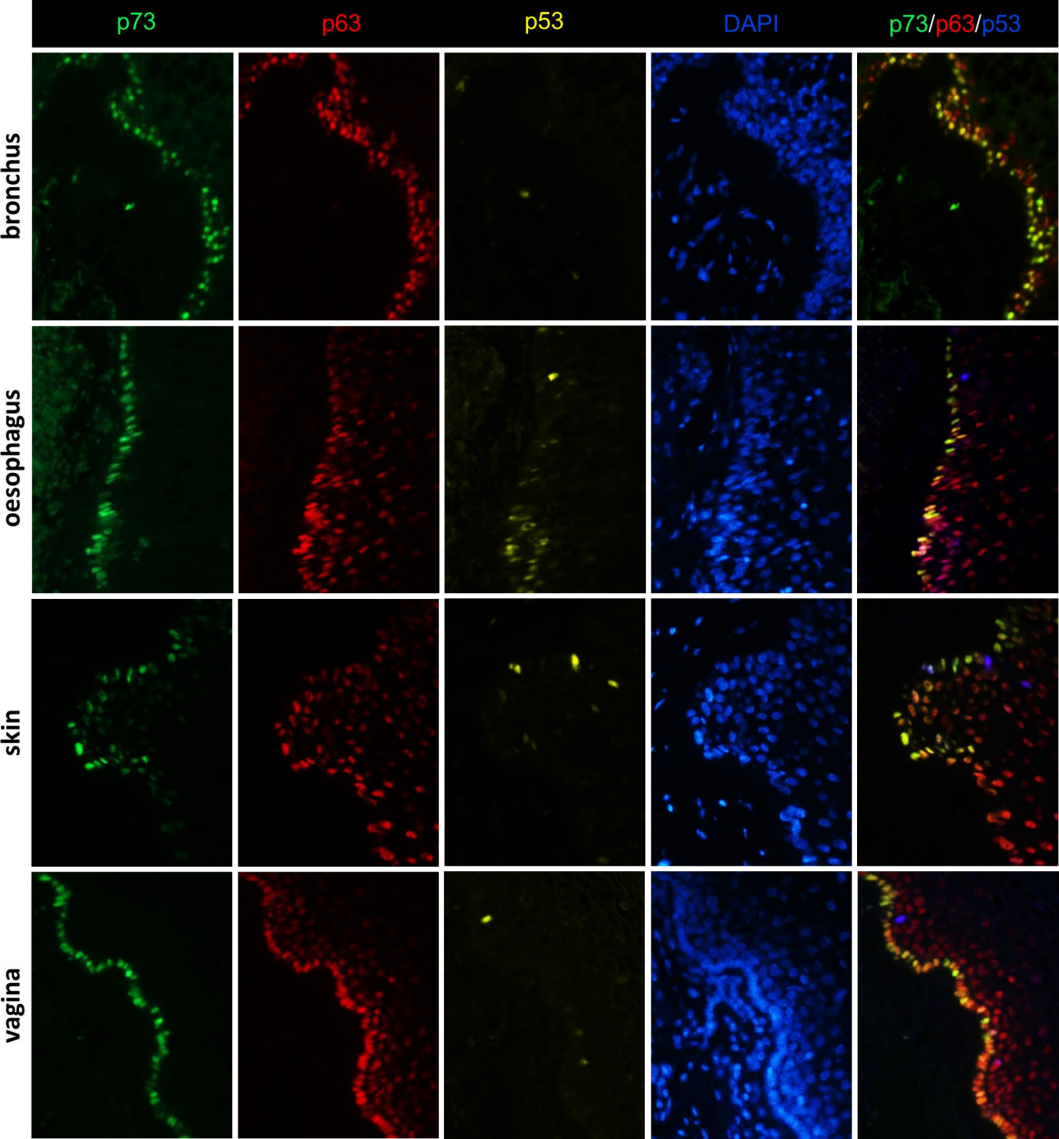
Triple immunofluorescent staining of p53, p63 and p73α in normal epithelial tissues. Formalin-fixed paraffin-embedded tissue sections were co-stained with antibodies to p73α (green), p63 (red) and p53 (yellow). Sections were counterstained with DAPI (blue). For easier visualisation, the DAPI staining channel was removed from the merged images on the right and the yellow channel of p53 was pseudo-coloured blue.

The above data indicate that a form of p73α that is not a TAp73 variant is located in nuclei in the basal layer of squamous epithelium. The highest p63 level is also seen in cells of the basal layer of squamous epithelium, gradually decreasing with cell differentiation and proximity to the surface of the epithelium^28^. Therefore, we investigated the co-localisation of p73 and p63 in squamous tissues, and of the third member of the family, p53. Formalin-fixed paraffin-embedded tissue sections were triple stained with antibodies to p73α (p73α-1.1 mouse), p63 (PANp63-6.1 mouse) and p53 (CM-1 rabbit). These data show that all p73α^+^ cells contain p63, although p63^+^ cells are not always p73α^+^, particularly p63^+^ parabasal cells. p53 does not show a regular pattern and is weak and confined to individual parabasal cells in normal squamous epithelia. In addition, p53^+^ cells lack either p63 or p73α (Fig. 6).

**Fig. 7:**
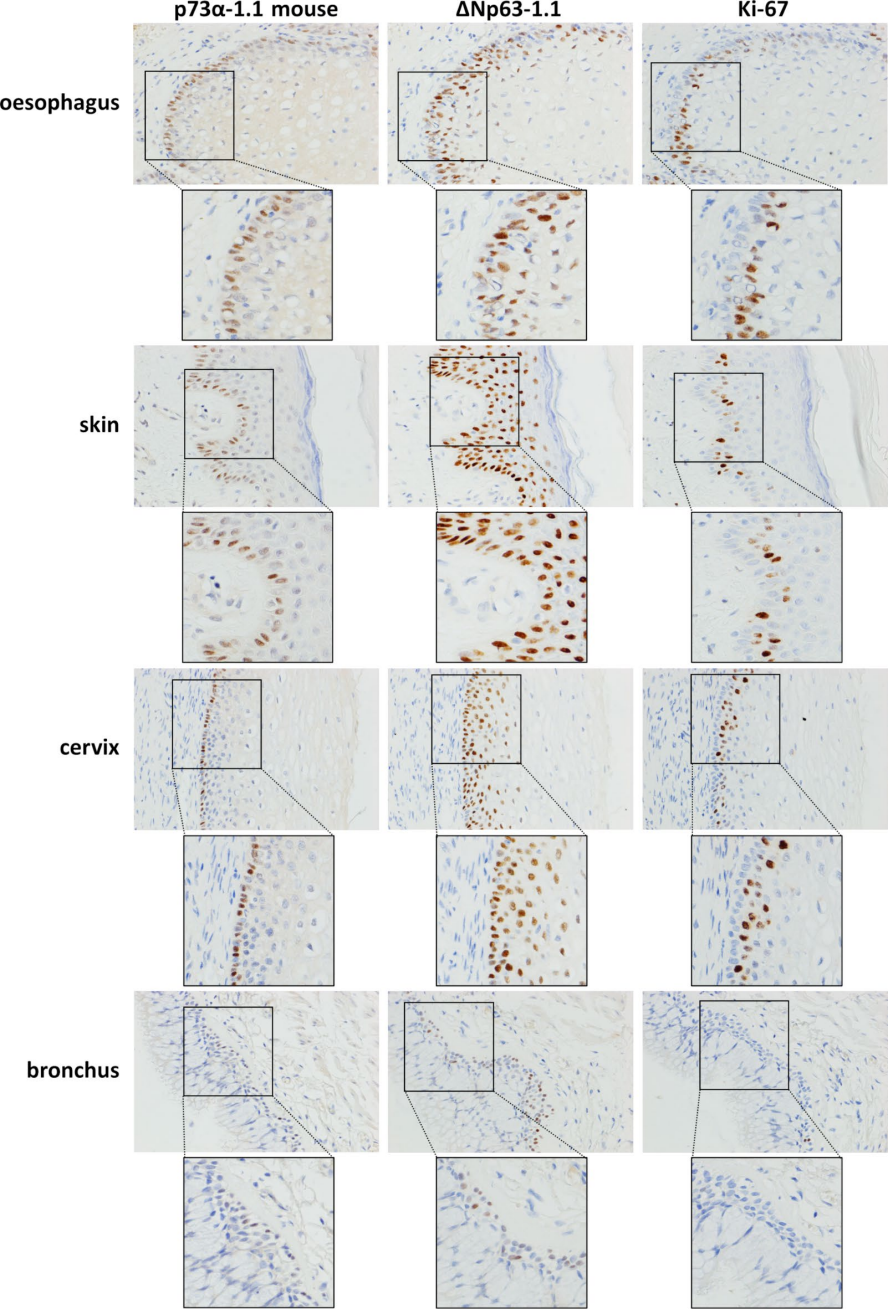
Immunohistochemical staining of p73α, ΔNp63 and Ki-67 in the indicated normal tissues. Positive staining is seen as brown reaction product. Nuclei are counterstained blue.

The distribution of p73α^+^ cells in basal squamous epithelial cells was also compared with the proliferation marker Ki-67. These data showed that ΔNp63^+^/p73α^+^ cells in the basal layer of squamous epithelium and bronchus are Ki-67^-^. In contrast, Ki-67^+^ cells are found in the ΔNp63^+^ but p73α^-^ suprabasal cells of squamous epithelium (Fig. 7).

**Table 1:** The distribution of p73 isoforms in normal tissues.

Tissue	ΔNp73	TAp73	p73α
Bone marrow	–	–	–
Brain	–	–	–
Breast	–	–	–
Bronchus	–	Ciliated epithelial cells	Ciliated and basal epithelial cells
Cervix	–	–	Basal epithelial cells
Duodenum	–	–	–
Fallopian tube	–	Ciliated epithelial cells	Ciliated and basal epithelial cells
Ileum	–	–	–
Kidney	–	–	–
Liver	–	–	–
Lymph node	–	–	–
Oesophagus	–	–	Basal epithelial cells
Ovary	–	–	–
Secretory endometrium	–	Ciliated epithelial cells	Ciliated epithelial cells
Skin	–	–	Basal epithelial cells
Testis	–	–	–
Thymus	–	–	Thymic epithelium
Tongue	–	–	Basal epithelial cells
Urothelium	–	–	Epithelial cells

### TAp73 and ΔTAp73α in cervical carcinoma

**Fig. 8:**
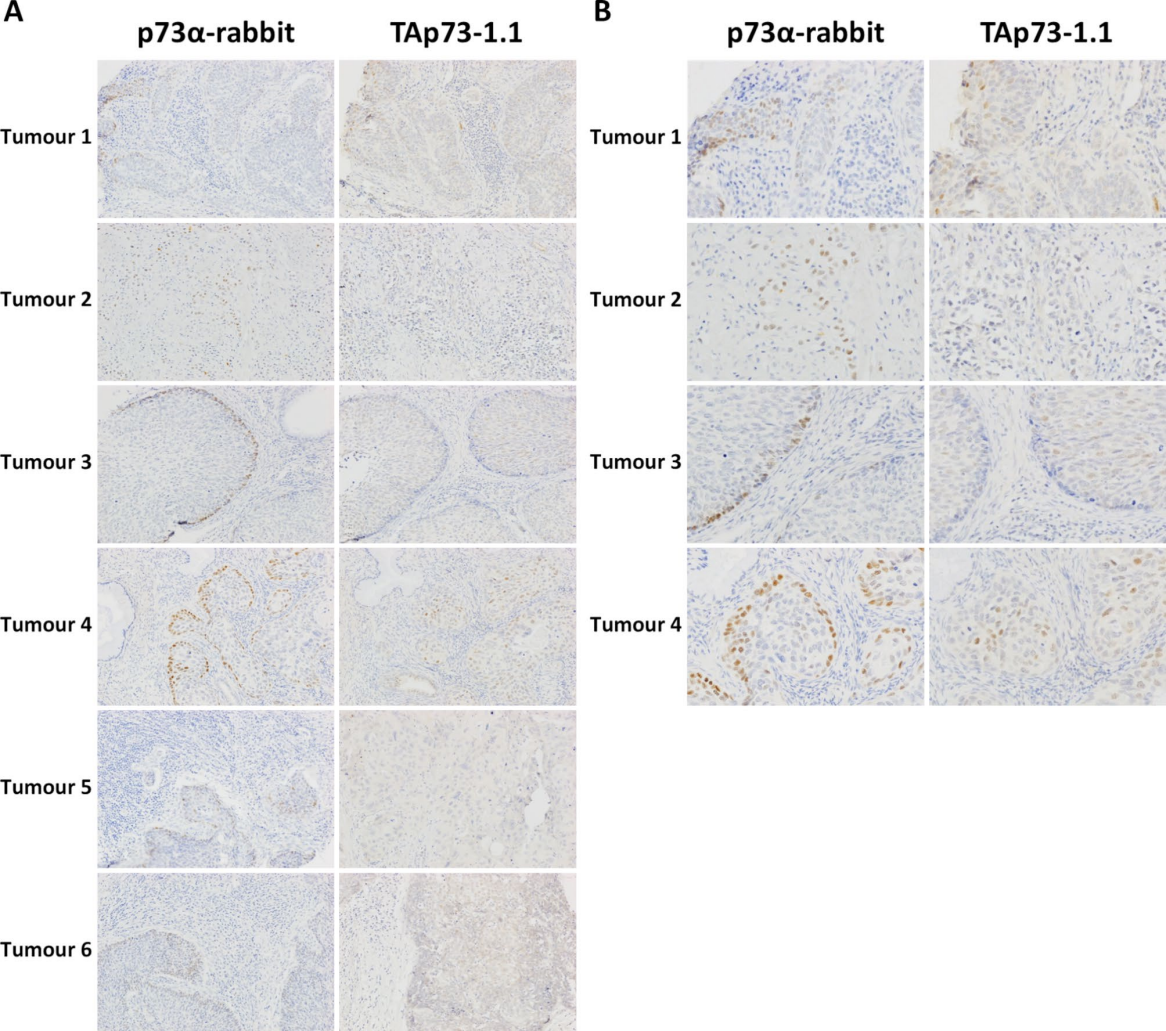
Immunohistochemical staining of cervical carcinoma. (**A**) Six different cervical tumours stained with affinity purified rabbit anti-p73α and mouse monoclonal TAp73-1.1 (brown). Sections were counterstained with haematoxylin (blue). (**B**) shows higher magnification images of the indicated tumours.

In view of the location of p73α in non-proliferative basal cells of normal squamous epithelium including the cervix, we investigated p73 isoform patterns in cervical cancer TMAs. p73α was present in the nuclei of cells in 49 of 62 squamous cell carcinomas, 5 of 14 adenocarcinomas and 1 of 2 neuroendocrine tumours. Cytoplasmic staining was not seen. Staining was predominantly located in a peripheral/basal pattern reminiscent of the distribution in normal cervical tissue in 19 squamous cell carcinomas and exhibited a more diffuse pattern in the remaining 30 (Fig. 8). TAp73 was present in the nuclei of tumour cells in 10 squamous cell carcinomas, 1 adenocarcinoma and 1 neuroendocrine tumour. TAp73^+^ cells were fewer in number than p73α^+^ cells and TAp73 was not found in peripheral/basal cells in any squamous cell carcinoma (Fig. 8). Statistically, more squamous cell carcinomas were p73α positive compared to adenocarcinomas (*p* = 0.003), whereas TAp73 was not associated with tumour type (*p* = 0.462) (Table 2). We also found an association between the peripheral/basal staining pattern of p73α in squamous cell carcinomas and tumour grade (*p* = 0.009 comparing grade 1 and 2 tumours to grade 3 tumours). There were no associations of p73α or TAp73 with disease progression, overall or disease-specific survival, lymph node invasion or Ki67 in either the whole cohort of tumours or in the squamous cell carcinoma subset of tumours (Table 2).

**Table 2:** Association of clinicopathological characteristics with p73α and TAp73 staining in cervical tumours.

	p73α			TAp73		
	Positive	Negative	*p*	Positive	Negative	*p*
Adenoca	5	9	**0.003**	1	12	0.462
SCC	49	13		10	48	
Progression = yes	7	5	0.493	2	9	1
Progression = no	47	18		10	51	
DoD = yes	6	3	1	1	8	1
DoD = no	49	20		11	53	
pN positive	19	7	0.797	4	22	1
pN negative	36	16		8	39	
^a^Ki67 ≥ 90%	23	8	0.793	7	22	0.338
Ki67 < 90%	28	12		5	33	

	Basal	Diffuse				
^b^grade 1/2	15	12	**0.009**			
Grade 3	4	18				

*AdenoCa* adenocarcinoma, *SCC* squamous cell carcinoma, *DoD* dead of disease, *pN* pathological regional lymph nodes.

Significant values are in bold.

^a^Different cut-offs for percentage of KI67^+^ tumour cells also showed no association with p73.

^b^Data are for squamous cell carcinomas only. Statistical evaluation was conducted using two-tailed Fisher exact probability tests.

## Discussion

The *TP73* gene can produce a number of different protein isoforms, each of which shares high homology with the two other members of the p53 family, p53 and p63, and have distinct functions and expression patterns. Within the range of their varying functional properties, TAp73 proteins have tumour-suppressive activities of apoptosis and growth inhibition due to the presence of the p53-like TA domain, while ΔTAp73 isoforms lack the TA domain and have oncogenic functions, acting as transdominant inhibitors of TAp73, TAp63 and p53^12,14,29–31^. Thus, there is a need for p53 family protein antibodies that do not cross-react with other family members, and for reagents that distinguish between the different protein isoforms. For the first of these two problems, many PAN-p73 antibodies (i.e. that recognise all p73 isoforms) bind both p63 and p73 or show a range of non-specific cross-reactions, including commercially available reagents. Similarly, most monoclonal PAN-p63 antibodies show cross-reaction with p73, including commercial and widely used p63 antibodies, and some also recognise p53^26,27^. For the second problem, reagents that lack isoform specificity are not able to identify whether the detected protein has tumour-suppressive activity (TA-isoforms) or is a pro-survival oncogenic protein (ΔTA-isoforms), hampering the interpretation of results using PAN-protein reagents. On the other hand, unlike the substantial degree of homologies in the central regions of p53, p63 and p73, the N-terminal and C-terminal sequences are more divergent^32^, reducing the chance of cross-recognition of antibodies that bind to these regions. However, antibody cross-reaction with unrelated proteins is not uncommon, emphasising the need for careful characterisation of novel reagents^33,34^. For example, the commonly used mouse monoclonal antibody 38C674.2 (IMG-313 A) to ΔNp73 binds to a non-specific band in Western blotting^26^ and shows widespread cytoplasmic staining in human tumour samples^35,36^ compared to the exclusively nuclear location seen for p73 using appropriately characterised reagents^18,37^.

Therefore, we developed and characterised novel antibodies against TAp73, ΔNp73 and p73α. The antibodies were extensively tested for cross-reactivity with the homologous proteins from p63 and p53 using Western blotting and immunohistochemistry prior to use. In addition, the precise epitopes of the monoclonal antibodies were determined by phage display technology, allowing their precise mapping and showing that the epitopes are not present in other p53 family proteins. Although the peptide sequence used for the production of p73α antibodies is also present in p73ε and p73ζ, data from next generation sequencing studies have demonstrated that p73α is the predominant variant in human and mouse cells and tissues^14,18^. p73α antibodies can therefore be considered as PAN-p73 reagents in most circumstances.

Using these antibodies, we stained a range of normal human tissues to investigate the distribution of ΔNp73, TAp73 and p73α. These data revealed that TAp73 is seen only in the nuclei of multiciliated cells, in keeping with induction of multiciliogenesis as its main or only function, and the lack of this isoform in p73^−/−^ mice can account for the majority of defects in these animals^21,23,38^. TAp73^+^ cells are also positive for p73α, indicating that multiciliated cells contain the TAp73α isoform. This highly restricted expression pattern of TAp73 indicates that it represents a specific marker of multiciliated cell differentiation in normal tissues, and immunohistochemical detection of total p73 identifies cryptic differentiation in endometrial carcinoma associated with good prognosis^39^. The ability to study the TAp73 isoform specifically should therefore act as a more reliable and specific marker of this process than total p73, and the use of TAp73 as a marker of differentiation in tumours derived from tissues with multiciliated cell differentiation warrants further study.

Our findings using antibodies that recognise p73α demonstrated that many more cells contain p73α than contain TAp73, particularly basal cells in squamous epithelia, indicating that these cells contain a ΔTAp73α variant. ΔNp73 was the first such variant described, arising from the P2 promoter in exon 3’ (Fig. 1)^15,19,40,41^, analogous to the mechanism used to produce ΔNp63^12^. In addition, ΔNp73 can be produced from *ΔN’p73* transcripts derived from the P1 promoter that splice exon 3 to exon 3’. *ΔNp73* and *ΔN’p73* mRNAs are reported to be overexpressed in many cancers and are linked with poor prognosis^42^, but it is important to note that studies investigating ΔNp73 and other p73 N-terminal isoforms rely on highly sensitive PCR methods able to identify extremely low mRNA levels (for example ~ 10 copies in an entire sample reaction^31^, equivalent to less than 1 copy/100 cells). By immunohistochemistry, we were unable to detect ΔNp73 in any of the tissues we studied. Although we cannot exclude the possibility that our ΔNp73 antibodies are sub-optimal for this purpose, none of the three independent monoclonal antibodies or an affinity purified rabbit polyclonal antibody were able to detect the protein despite showing good performance by Western blotting and immunohistochemistry of FFPE cells, and being used at higher concentrations than those used for the other reagents produced here. These findings therefore suggest that ΔNp73 is a minor p73 protein variant in human tissues, which agrees with recent data that ΔTAp73 variants are produced mainly from transcripts derived from a promoter immediately upstream of exon 4^18^, with the first in-frame ATG site in exon 4 used for translation^18^. Alternative translation initiation of *TAp73* transcripts at the same ATG within exon 4 has also been described^43^. Thus, it will be important to identify the precise nature and origin of p73α variant proteins that lack the TA domain. ΔTAp73 isoforms formed by alternative translation initiation at the ATG within exon 4 lack unique N-terminal amino acid sequences and it will be difficult to produce antibodies that are specific for these isoforms. However, in the absence of such reagents, a combination of p73α positivity with negative TAp73 staining indicates the presence of ΔTAp73 proteins derived from any potential mechanism.

The location of p73α^+^/TAp73^−^ cells, restricted to the basal layer of squamous epithelium and bronchus, suggests a role in the stem cell populations of these tissues. These p73α^+^ cells are also ΔNp63^+^, whereas ΔNp63 is also present in parabasal cells. Given that p63 is known to regulate squamous epithelial cell commitment, differentiation and stem cell activities^44,45^, and that p73 forms heterotetramers with p63^46^, these data imply that the presence of both ΔTAp73 and ΔNp63 is important for maintaining the non-proliferative stem cell (reserve) population of squamous epithelia, and lack of p73 in the continued presence of ΔNp63 allows proliferation and early stage differentiation. Further work will be required to delineate the effects of ΔTAp73 in the presence of p63 in regulating normal squamous cells, and in squamous cell carcinomas.

In our dataset of cervical tumours, we identified an association of p73α with squamous cell carcinomas compared to adenocarcinomas. We also identified distinct patterns of ΔTAp73α in squamous carcinomas, with basal patterns of p73α expression more common in lower grade tumours. This implies that p73 in basal tumour cells indicates an appropriate regulation of p73 that is lost as tumours progress. In addition, although TAp73 is absent in normal cervical tissue, some cervical carcinomas showed positive staining, with the ΔTAp73α basal/peripheral tumour cells not being TAp73^+^. This situation is similar to p63, where TAp63 is not present in normal adult squamous epithelium but is seen in a minor proportion of tumour cells in some squamous cell carcinomas and other tumours that express predominantly ΔNp63, with accompanying clinical associations^27,47,48^. It is therefore likely that *TP73* isoform regulation in cancer cells is less stringent than in normal cells, and further studies will be necessary to elucidate *TP73* isoform regulation and the clinicopathologic characteristics of ΔTAp73 and TAp73 in squamous cell carcinomas and other tumour types.

In conclusion, we have produced novel monoclonal and polyclonal antibodies to p73 isoforms that do not cross-react with other p53 family members and are applicable to Western blotting and immunohistochemistry of routine clinical FFPE tissue sections. These antibodies provide reliable tools that will be valuable for studying the functional properties of individual p73 isoforms, including protein-protein interactions, for example using proximity ligation assays^49^, and for DNA binding studies. Our data indicate that TAp73 is a marker of commitment to multiciliated cell differentiation in normal epithelial tissues, with implications for identifying the differentiation status of cells in tumours derived from tissues that contain multiciliated cell types, including endometrial carcinomas^50^ and ovarian serous cancers that originate in the fallopian tube^51^. In addition, we show that non-proliferative basal epithelial cells contain both ΔTAp73 and ΔNp63, whereas cells in the proliferative compartment express only ΔNp63, suggesting roles for ΔTAp73 in combination with ΔNp63 in regulating proliferation/and or cellular differentiation in these tissues and their tumours.

## Materials and methods

### Development of isoform-specific p73 antibodies

Antibodies were developed by Moravian-Biotechnology spol. s r.o. (Brno, Czech Republic), who performed immunisations, serum collection and preparation of mouse hybridomas. The animal experiments were performed in the accredited animal facilities of the Veterinary Research Institute in Brno after approval by the Animal Welfare Committee of the Ministry of Agriculture of the Czech Republic (permits MZe No. 2188 - rabbits and MZe No. 2187 - mice). The animal care protocol for this experiment followed the Czech Guidelines for Animal Experimentation. TAp73 antibody was prepared against purified recombinant protein containing the full TAD sequence of human TAp73α. Antibodies to p73α were produced in rabbits or mice immunised with the peptide CKARKQPIKEEFTEAEIH conjugated to keyhole limpet haemocyanin at the N-terminal cysteine. This peptide represents the C-terminal 18 amino acids of human p73α (p73ε and p73ζ share the same C-terminal amino acid sequence). The corresponding mouse and rat sequences share high homology; (CKSRKQPIKEEFTETESH). Polyclonal p73α rabbit serum was affinity purified using the same peptide conjugated to beads using SulfoLink Immobilization (Thermo Scientific Pierce, MA, USA). Anti-ΔNp73 antibodies were produced in rabbits and mice using the peptide MLYVGDPARHLATAQ, representing the 16 amino acids at the N-terminus of human ΔNp73 (including the 14 amino acids sequence unique to ΔNp73 and 2 amino acids that are common with all isoforms, similar to the approach used to produce ΔNp63-specific antibodies^27,52,53^). The same amino acid sequence is present in ΔN’p73. Rabbit ΔNp73 sera were affinity purified against the immunising peptide conjugated to beads using SulfoLink Immobilization.

### Cloning and recombinant protein preparation

Recombinant p73 TAD protein was prepared using Gateway recombination cloning technology and vectors (Thermo Fisher Scientific, MA, USA). The TAD entry clone sequence was prepared in two PCR reactions. First, TAp73-TEV-GW forward primer containing the tobacco etch virus (TEV) protease sequence and TAp73-GW-reverse primer (Supplementary Table S1 online) were used to amplify pcDNA3-TAp73α that contains full-length human TAp73α cDNA. In the second PCR, attB1 and attB2 recombination sequences were added using universal-attB1-TEV-GW forward primer and universal-attB2-GW reverse primer (Supplementary Table S1 online). The final Gateway entry clone containing the TAp73 TA domain sequence was cloned into pDONR 221 and then into the expression vector pDEST15 containing an N-terminal His_6_-GST tag. The resulting clones were sequenced (Eurofins genomics, Benesov, Czech Republic).

The TAD protein was expressed in *E. coli* BL21 (DE3) RIPL cells. Cells were grown in LB medium at 37 °C until OD_600_ reached 0.5 and protein expression was induced by adding 1 mM isopropyl β-d-thiogalactopyranoside (IPTG). Bacteria were collected after 4 h at 30 °C and centrifuged at 6000 g for 10 min at 4 °C. The bacterial pellet was resuspended in lysis buffer A [PBS pH 7.4; 0.5% Triton X-100; lysozyme (1 mg/ml); 1 mM phenylmethylsulfonyl fluoride (PMSF)] on ice. The bacterial suspension was sonicated on ice for 15 min (10 s ON, 50 s OFF) at 40% amplitude and then centrifuged. The pellet containing inclusion bodies with insoluble proteins was resuspended in lysis buffer B (50-mM Tris pH 8,5; 100 mM NaCl; 2% sodium lauroyl sarcosinate) and centrifuged again to remove non-solubilised protein and debris. The supernatant was incubated with Ni-NTA Agarose (Qiagen, Germany) at 4 °C for 24 h. Ni-NTA agarose with bound TAD was washed seven times with 5 ml lysis buffer B, and the TAD protein was eluted with 250 mM imidazole in 50 mM Tris pH 8.5; 100 mM NaCl; 2% sodium lauroyl sarcosinate.

### Cell culture and transfection

H1299 cell line (human non-small cell lung carcinoma) was obtained from the American Type Culture Collection (CRL-5803, ATCC, Manassas, VI, USA). H1299 cells were grown in Dulbecco’s modified essential medium (DMEM) supplemented with 10% fetal bovine serum, penicillin/streptomycin, and pyruvate (Invitrogen, CA, USA) at 37 °C in a humidified atmosphere with 5% CO_2_. The cells were grown on 10 cm diameter plates up to 70% confluency and transfected with 8 µg of plasmid vectors containing sequences coding for p53, ΔNp63α, TAp63α, ΔNp73α, TAp73α, TAp73β or TAp73γ, using polyethyleneimine at a final concentration of 2.1 µg/ml. Cells were harvested 24 h after transfection.

### Preparation of paraffin-embedded cell culture blocks

H1299 cells were trypsinised, rinsed in PBS and fixed in 4% paraformaldehyde in PBS overnight at room temperature (RT). After fixation, cells were washed in PBS and centrifuged at 1000 g for 1 min at RT. The pellet was resuspended in 200 µl 1.5% melted agarose (SERVA, Germany) in a water bath at 42 °C. Cells in agarose were processed and embedded in paraffin using the same protocol as for tissue processing.

### Immunohistochemistry

Anonymised excess formalin-fixed paraffin-embedded (FFPE) tissues were obtained from the Department of Pathology at Masaryk Memorial Cancer Institute. Patients provided informed consent for the use of their tissues for research. These samples included a range of histologically normal tissues and tissue microarrays (TMA) of cervical carcinomas. The study complied with the Declaration of Helsinki and was approved by the MMCI ethical committee (approval number MOU 205 662). Sections (4 μm) on glass slides were dried overnight, deparaffinised and rehydrated. Endogenous peroxidase activity was blocked by incubation in 3% H_2_O_2_ in PBS pH 7.4 for 5 min. Antigen retrieval was performed by boiling in 1mM EDTA pH 8.0 for 20 min followed by 20 min cooling. Sections were blocked with antibody diluent solution (DAKO, Agilent, CA, USA) and incubated for 24 h at 4 °C with primary antibodies diluted in the same buffer. After washes in PBS, sections were incubated for 1 h at RT with horseradish peroxidase-conjugated secondary antibodies (DAKO Envision+) and washed three times in PBS. EnVision FLEX DAB+ (DAKO) was used to visualise staining and haematoxylin was used to counterstain cell nuclei. Sections were dehydrated and mounted in Entellan (Merck Millipore, Germany) for light microscopy. Ki-67 staining used MIB-1 (M7240, DAKO).

### Western blotting

Adherent cells were washed with ice-cold PBS pH 7.4 and harvested into lysis buffer (50 mM Tris/HCl pH 8.0, 150 mM NaCl, 1% NP40, 50 mM NaF, 5 mM EDTA, 1 mM PMSF) with protease inhibitor cocktail (Pierce Biotechnology, IL, USA). The cell suspension was left for 30 min on ice with regular vortexing, and centrifuged at 20 000 g for 30 min at 4 °C. Protein concentration was determined by Bradford assay and equal amounts of proteins were separated by electrophoresis on 10% polyacrylamide gels and transferred onto nitrocellulose membranes. Membranes were blocked in 5% non-fat dried milk in PBS pH 7.4 with 0.1% Tween 20 (PBST) before incubation for 24 h at 4 °C with primary antibodies diluted in blocking buffer. After washing in PBST, membranes were incubated with HRP-conjugated anti-mouse or anti-rabbit antibodies (Agilent, CA, USA) diluted in blocking buffer for 1 h at RT. Enhanced chemiluminescence (ECL; Amersham Pharmacia Biotech, UK) was used for detection and membranes were exposed to X-ray film.

### Epitope mapping by phage display

All buffers were filtered through a 0.22 μm filter before use. Protein G magnetic beads (Invitrogen, CA, USA) were washed three times in PBST and resuspended in a ratio of 1:10 in PBST. 100 µg purified antibody was added to 100 µl bead suspension in a polypropylene 96-well plate and shaken for 1 h at RT. Beads were washed twice in PBST containing 1 mg/ml BSA, transferred to a new plate and washed once in the same buffer. Subsequently, beads were resuspended in 100 µl PBST/BSA. Ph.D.™-12 Phage Display Peptide Library (New England Biolabs, MA, USA) was diluted 1:50 in PBST/BSA. Beads were incubated with 100 µl diluted phage library (2 × 10^10^ pfu per sample), shaken for 1 h at RT and washed twice each in PBST and TBS (500 mM NaCl, 50 mM Tris pH 8). Washed beads were resuspended in PBST, transferred to a new plate and washed once more in PBS containing 0.05% Tween20. Antibodies and bound phages were eluted in 50 µl 0.1 M glycine pH 3 and neutralised with 8 µl 1 M Tris pH 8. A DNA library of the eluted phages was prepared and amplified in three sets of PCR (Supplementary Tables S2, S3, S4 online) using Herculase II fusion DNA polymerase (Agilent, CA, USA). Each PCR product was size selected and pre-cleaned using SPRI beads (Beckman Coulter, IN, USA). The phage library was sequenced using the Illumina Nextseq 550 system (Illumina, CA, USA). Reads were first subjected to trimming, and unique 12 amino acid sequences were analysed and aligned to human p73 protein sequences. In parallel, a similar analysis was performed using the Hammock software^54^, which employs a hidden Markov model-based clustering algorithm. Outputs were visualised as a WebLogo, highlighting a specific binding motif.

### Triple immunofluorescent staining

FFPE tissue sections were deparaffinised and rehydrated, endogenous peroxidase was blocked in 3% H_2_O_2_ for 5 min and antigen retrieval was performed by boiling in 1 mM EDTA, pH 8.0. After incubation with blocking solution (10% goat serum, Invitrogen, Thermo Fisher Scientific, MA, USA), mouse monoclonal antibody p73-1.1 (recognising p73α) was applied overnight at 4 °C. HRP-conjugated anti-mouse secondary antibody (Invitrogen, Thermo Fisher Scientific, MA, USA) was applied for 1 h at RT and Alexa Fluor 488 tyramide working solution (Invitrogen, Thermo Fisher Scientific, MA, USA) was applied for 10 min at RT. Afterwards, Reaction Stop Reagent (Invitrogen, Thermo Fisher Scientific, MA, USA) was applied for 1 min. Tissues were then incubated with blocking solution for 1 h at RT and primary antibodies against p63 (mouse monoclonal PAN-p63 6.1, in-house) and p53 (rabbit polyclonal antibody CM-1, in-house) were applied together at 4 °C overnight. The next day, poly-HRP-conjugated secondary anti-rabbit (Invitrogen, Thermo Fisher Scientific, MA, USA) together with secondary Alexa Fluor 647 conjugated anti-mouse (Southern Biotech, AL, USA) were applied for 1 h at RT. Alexa Fluor 555 tyramide working solution (Invitrogen, Thermo Fisher Scientific, MA, USA) was applied for 10 min and Reaction Stop Reagent was applied for 1 min. Cells were mounted with ProLong Glass Antifade Mountant with NucBlue Stain (Invitrogen, Thermo Fisher Scientific, MA, USA) and images were taken by fluorescent microscopy (Eclipse Ti-E, Nikon, Japan).

## Electronic supplementary material

Below is the link to the electronic supplementary material.

Supplementary Material 1

## Data Availability

The nucleotide sequencing datasets generated during the current study are available in the European Nucleotide Archive (https://www.ebi.ac.uk/ena/browser/home), under study accession number PRJEB80529. All other data generated or analysed during this study are included in this published article and its supplementary information files.

## Abbreviations

DBD: DNA binding domain
FFPE: formalin-fixed paraffin-embedded
OD: oligomerisation domain
RT: room temperature
TAD: transactivation domain
