# Supplementary material for: TAp73 and ΔTAp73 isoforms show cell-type specific distributions and alterations in cancer
Source: Sci Rep. 2024 Dec 2;14:29949. doi: 10.1038/s41598-024-80927-9 (PMC11612387; doi:10.1038/s41598-024-80927-9)
Supplement: Supplementary file 1 — Supplementary Material 1 [file 41598_2024_80927_MOESM1_ESM.pdf]

Supplementary data for:

TAp73 and ΔTAp73 isoforms show cell-type specific distributions and alterations in cancer

Vaclav Hrabal, Michaela Stenckova, Filip Zavadil Kokas, Petr Muller, Rudolf Nenutil, Borivoj Vojtesek, Philip J. Coates

Supplementary Figure S1 Original images used for Figure 2

Figure 2A

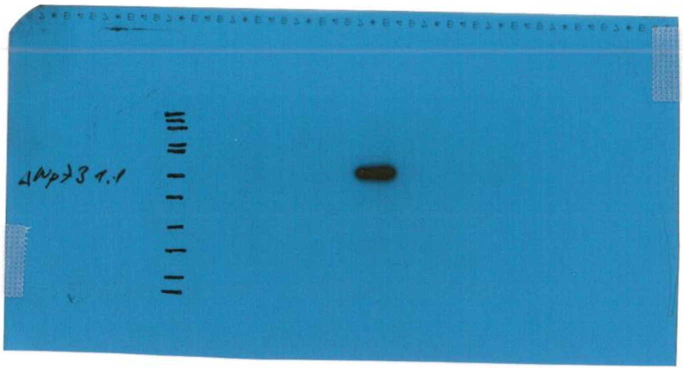

Figure 2B

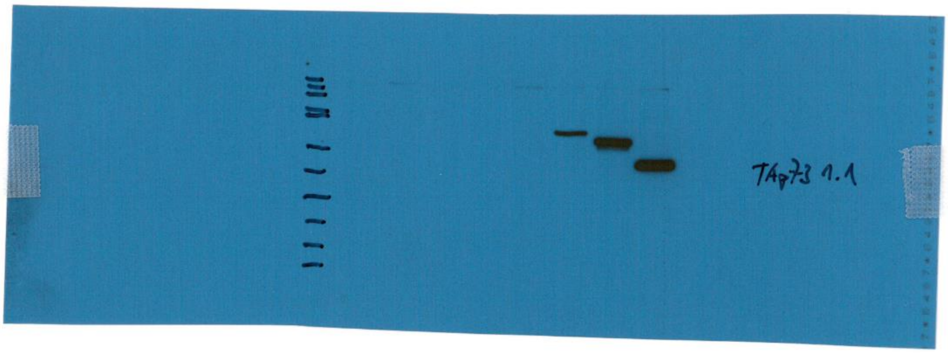

Figure 2C

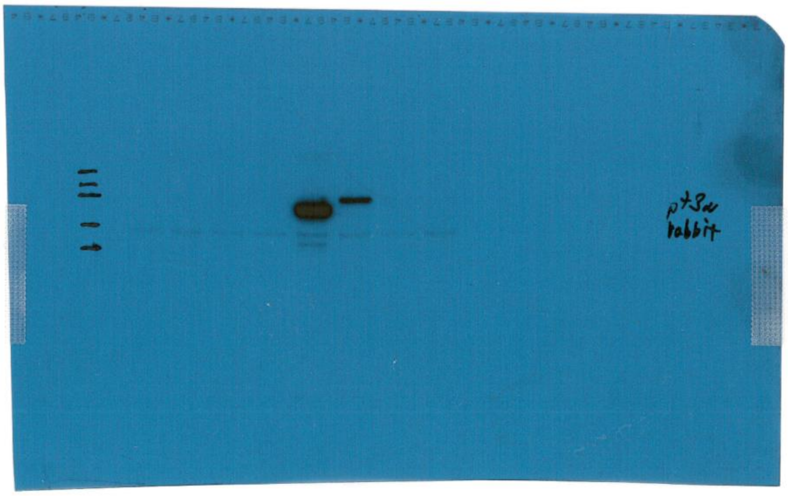

Figure 2D

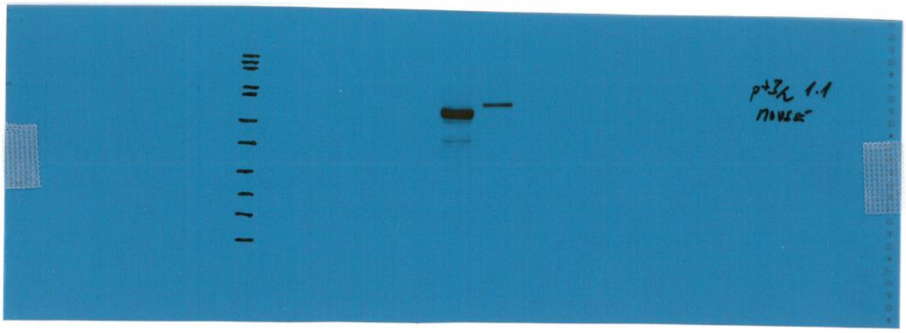

Figure 2E

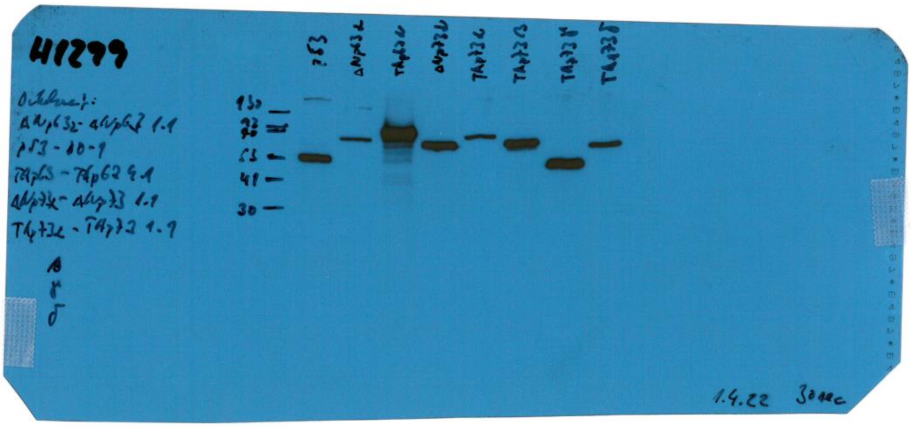

Supplementary Figure S2 Immunohistochemical staining of normal skin tissue with different clones of p73α monoclonal antibodies.

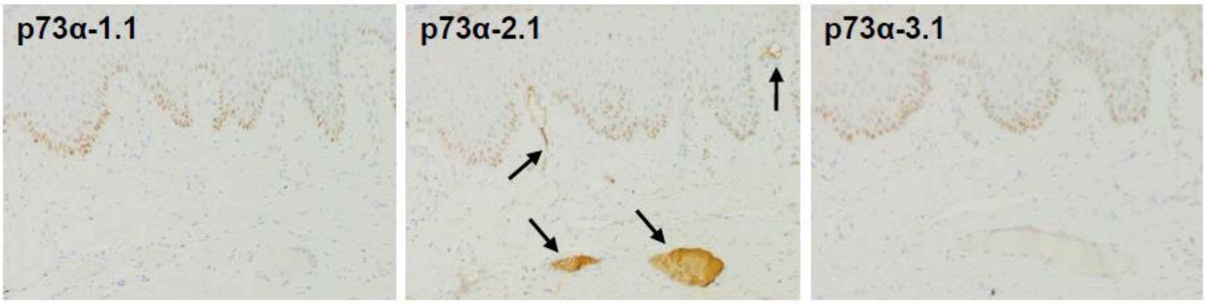

Supplementary Figure S3 Immunohistochemical staining of normal human tissues that are not p73-positive.

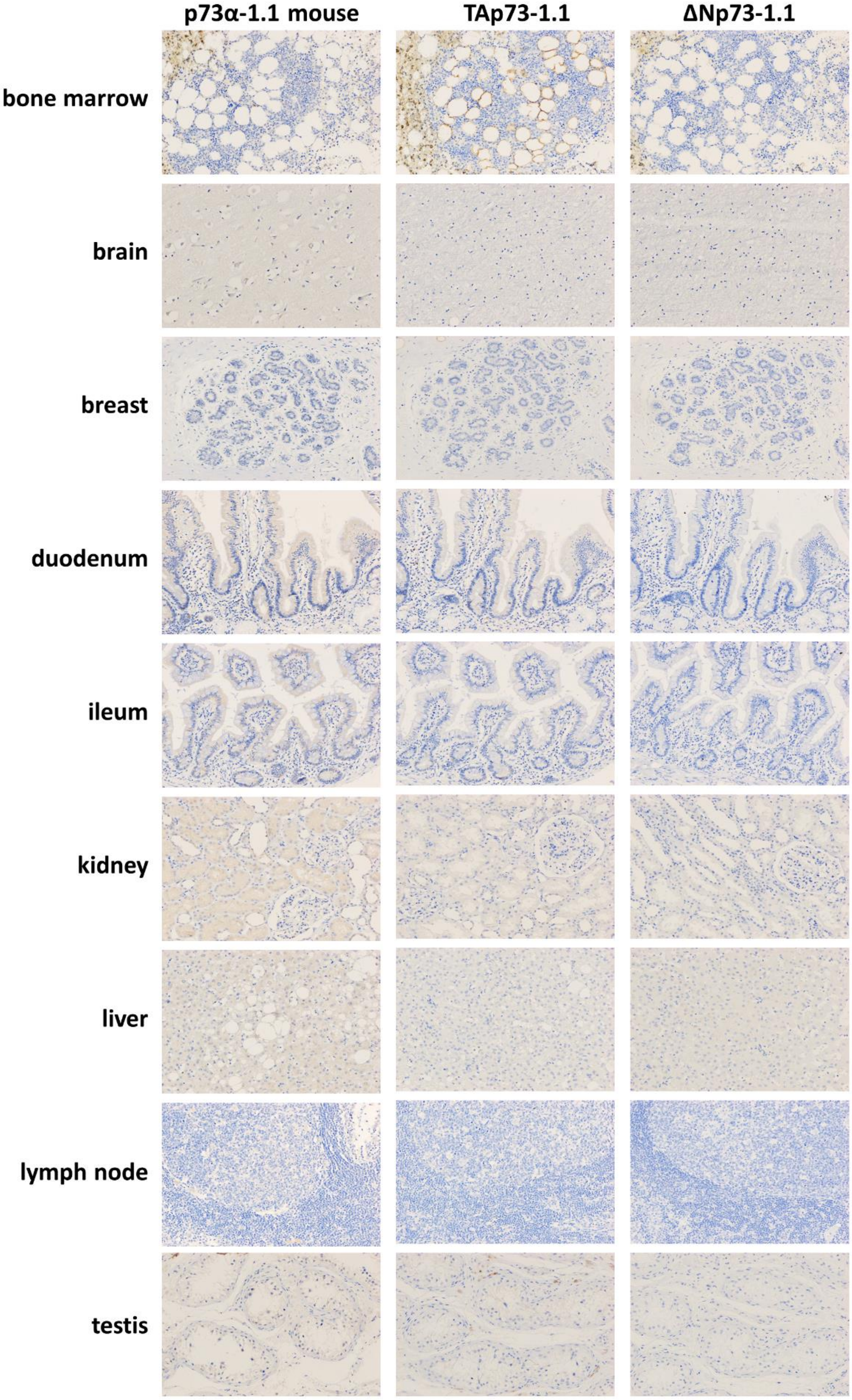

Supplementary Table S1 Primer sequences

**Primers**	**Sequences**
TAp73-TEV-GW forward	5´-GGCTCTGAGAACCTGTACTTCCAGAGCATGGCCCAGTCCACCGCCAC-3´
TAp73-GW reverse	5´-GTACAAGAAAGCTGGGTTCATGACAGATGTAGTCATGCCCTCC-3´
universal-attB1-TEV-GW forward	5´-GGGGACAAGTTTGTACAAAAAAGCAGGCTTCGGCTCTGAGAACCTGTACTTC-3´
universal-attB2-GW reverse	5´-GGGGACCACTTTGTACAAGAAAGCTGGGTT-3´
Phage-PCR forward	5´-CGTGGGCGATGGTTGTTGTC-3´
Phage-PCR reverse	5´-TAAGTGCCGTCGAGAGGGTTGATA-3´
Read1-GSP-P3 forward	5´-ACACTCTTTCCCTACACGACGCTCTTCCGATCTTTCGCAATTCCTTTAGTGGT-3´
Read2-GSP-P3 reverse	5´- GTGACTGGAGTTCAGACGTGTGCTCTTCCGATCTGGGATTTTGCTAAACAACTTTCA-3´
Index primer i501	5´- AATGATACGGCGACCACCGAGATCTACACTATAGCCTACACTCTTTCCCTACACGAC-3´
Index primer i502	5´- AATGATACGGCGACCACCGAGATCTACACATAGAGGCACACTCTTTCCCTACACGAC-3´
Index primer i503	5´- AATGATACGGCGACCACCGAGATCTACACCCTATCCTACACTCTTTCCCTACACGAC-3´
Index primer i504	5´- AATGATACGGCGACCACCGAGATCTACACGGCTCTGAACACTCTTTCCCTACACGAC-3´
Index primer i505	5´- AATGATACGGCGACCACCGAGATCTACACAGGCGAAGACACTCTTTCCCTACACGAC-3´
Index primer i506	5´- AATGATACGGCGACCACCGAGATCTACACTAATCTTAACACTCTTTCCCTACACGAC-3´
Index primer i507	5´- AATGATACGGCGACCACCGAGATCTACACCAGGACGTACACTCTTTCCCTACACGAC-3´
Index primer i508	5´- AATGATACGGCGACCACCGAGATCTACACGTACTGACACACTCTTTCCCTACACGAC-3´
Index primer i701	5´- CAAGCAGAAGACGGCATACGAGATCGAGTAATGTGACTGGAGTTCAGACGTGT-3´
Index primer i702	5´- CAAGCAGAAGACGGCATACGAGATTCTCCGGAGTGACTGGAGTTCAGACGTGT-3´
Index primer i703	5´- CAAGCAGAAGACGGCATACGAGATAATGAGCGGTGACTGGAGTTCAGACGTGT-3´
Index primer i704	5´- CAAGCAGAAGACGGCATACGAGATGGAATCTCGTGACTGGAGTTCAGACGTGT-3´
Index primer i705	5´- CAAGCAGAAGACGGCATACGAGATTTCTGAATGTGACTGGAGTTCAGACGTGT-3´
Index primer i706	5´- CAAGCAGAAGACGGCATACGAGATACGAATTCGTGACTGGAGTTCAGACGTGT-3´
Index primer i707	5´- CAAGCAGAAGACGGCATACGAGATAGCTTCAGGTGACTGGAGTTCAGACGTGT-3´
Index primer i708	5´- CAAGCAGAAGACGGCATACGAGATGCGCATTAGTGACTGGAGTTCAGACGTGT-3´
Index primer i709	5´- CAAGCAGAAGACGGCATACGAGATCATAGCCGGTGACTGGAGTTCAGACGTGT-3´
Index primer i710	5´- CAAGCAGAAGACGGCATACGAGATTTCGCGGAGTGACTGGAGTTCAGACGTGT-3´
Index primer i711	5´- CAAGCAGAAGACGGCATACGAGATGCGCGAGAGTGACTGGAGTTCAGACGTGT-3´
Index primer i712	5´- CAAGCAGAAGACGGCATACGAGATCTATCGCTGTGACTGGAGTTCAGACGTGT-3´

Supplementary Table S2 Phage display library PCR 1

PCR 1						
**Reagents**			**Conditions**		
**Reagents**	**Volume**		**Temperature**	**Time**	**Number of cycles**
5x Herculase II reaction buffer	10 µl		95 °C	5 minutes	1
dNTP mix (25 mM each)	0.5 µl		95 °C	15 seconds	30
Betain (5 M)	5 µl		54 °C	20 seconds	
Phage-PCR forward primer (100 µM)	0.2 µl		70 °C	20 seconds	
Phage-PCR reverse primer (100 µM)	0.2 µl				
Herculase II fusion DNA polymerase	0.5 µl				
Template (eluted phages)	33.6 µl				

Supplementary Table S3 Phage display library PCR 2

PCR 2					
**Reagents**			**Conditions**		
**Reagents**	**Volume**		**Temperature**	**Time**	**Number of cycles**
5x Herculase II reaction buffer	10 µl		95 °C	5 minutes	1
dNTP mix (25 mM each)	0.5 µl		95 °C	15 seconds	20
Betain (5 M)	5 µl		55 °C	30 seconds	
Read1-GSP-P3 forward primer (100 µM)	0.2 µl				
Read2-GSP-P3 reverse primer (100 µM)	0.2 µl				
Herculase II fusion DNA polymerase	0.5 µl				
H_2O	23.6 µl				
PCR 1 product template (5 ng)	10 µl				

Supplementary Table S4 Phage display library PCR 3

PCR 3					
**Reagents**			**Conditions**		
**Reagents**	**Volume**		**Temperature**	**Time**	**Number of cycles**
5x Herculase II reaction buffer	10 µl		95 °C	1 minute	1
dNTP mix (25 mM each)	0.5 µl		95 °C	15 seconds	8
Betain (5 M)	5 µl		55 °C	20 seconds	
Index primer i5.. (10 µM)	2 µl				
Index primer i7.. (10 µM)	2 µl				
Herculase II fusion DNA polymerase	0.5 µl				
H_2O	20 µl				
PCR 2 product template (25 ng)	10 µl				
